# Supplementary material for: The Systems Biology Research Tool: evolvable open-source software
Source: BMC Syst Biol. 2008 Jun 29;2:55. doi: 10.1186/1752-0509-2-55 (PMC2446383; doi:10.1186/1752-0509-2-55)
Supplement: Additional file 1 — SBRT Archive. An archive of the current version of the Systems Biology Research Tool. [file 1752-0509-2-55-S1.zip › sbrt-1.4.0/doc/developers_guide/api/sbrt/kernel/fba/proc/package-tree.html]

sbrt.kernel.fba.proc Class Hierarchy


|  |  |  |
| --- | --- | --- |
| **Overview** \| **Package** \| Class \| Use \| **Tree** \| **Deprecated** \| **Index** \| **Help** | |  |
| **PREV**   **NEXT** | **FRAMES**    **NO FRAMES**     **All Classes** |  |


---


## Hierarchy For Package sbrt.kernel.fba.proc

**Package Hierarchies:**: All Packages

---

## Class Hierarchy

- java.lang.Object
  - sbrt.kernel.fba.proc.**FbaProcess**<R> (implements sbrt.kernel.KernelProcess<T>)
    - sbrt.kernel.fba.proc.**CatalystDeletionProcess** (implements sbrt.kernel.MonitoredProcess<R>)- sbrt.kernel.fba.proc.**CdHarFluxVectorProcess** (implements sbrt.kernel.MonitoredProcess<R>)- sbrt.kernel.fba.proc.**CvCatalystDeletionProcess** (implements sbrt.kernel.MonitoredProcess<R>)- sbrt.kernel.fba.proc.**CvFbaOptimizationExperiment** (implements sbrt.kernel.MonitoredProcess<R>)- sbrt.kernel.fba.proc.**CvFbaOptProcess**<R>
              - sbrt.kernel.fba.proc.**CvFluxIntervalProcess** (implements sbrt.kernel.MonitoredProcess<R>)- sbrt.kernel.fba.proc.**SvCdHarFluxVectorProcess** (implements sbrt.kernel.MonitoredProcess<R>)- sbrt.kernel.fba.proc.**SvInitialPointProcess** (implements sbrt.kernel.MonitoredProcess<R>)
              - sbrt.kernel.fba.proc.**CvObjectiveFunctionProcess** (implements sbrt.kernel.MonitoredProcess<R>)- sbrt.kernel.fba.proc.**CvRxnDeletionProcess** (implements sbrt.kernel.MonitoredProcess<R>)- sbrt.kernel.fba.proc.**CycleIdProcess** (implements sbrt.kernel.MonitoredProcess<R>)- sbrt.kernel.fba.proc.**ExtremeCurrentProcess** (implements sbrt.kernel.MonitoredProcess<R>)- sbrt.kernel.fba.proc.**FbaOptimizationProcess**- sbrt.kernel.fba.proc.**FbaSolverProcess**- sbrt.kernel.fba.proc.**FluxActivityProcess** (implements sbrt.kernel.MonitoredProcess<R>)- sbrt.kernel.fba.proc.**FluxCapProcess** (implements sbrt.kernel.MonitoredProcess<R>)- sbrt.kernel.fba.proc.**FluxIntervalProcess** (implements sbrt.kernel.MonitoredProcess<R>)- sbrt.kernel.fba.proc.**FluxomeReducerProcess**<T> (implements sbrt.kernel.MonitoredProcess<R>)- sbrt.kernel.fba.proc.**FluxPlasticityProcess** (implements sbrt.kernel.MonitoredProcess<R>)- sbrt.kernel.fba.proc.**InitialPointGenerator**- sbrt.kernel.fba.proc.**NetworkInfoProcess**- sbrt.kernel.fba.proc.**ObjectiveFunctionAnalysis** (implements sbrt.kernel.MonitoredProcess<R>)- sbrt.kernel.fba.proc.**RandomConstraintsGeneratorV1** (implements sbrt.kernel.MonitoredProcess<R>)- sbrt.kernel.fba.proc.**RandomEnvGeneratorV1** (implements sbrt.kernel.MonitoredProcess<R>)- sbrt.kernel.fba.proc.**RandomObjectiveGenerator** (implements sbrt.kernel.MonitoredProcess<R>)- sbrt.kernel.fba.proc.**RxnDeletionProcess** (implements sbrt.kernel.MonitoredProcess<R>)- sbrt.kernel.fba.proc.**StoichVariationProcess** (implements sbrt.kernel.MonitoredProcess<R>)
    - sbrt.kernel.fba.proc.**FluxActivityProcess.Results**- sbrt.kernel.fba.proc.**FluxPlasticityProcess.Results**- sbrt.kernel.fba.proc.**NetworkInfoProcess.Results**

---


|  |  |  |
| --- | --- | --- |
| **Overview** \| **Package** \| Class \| Use \| **Tree** \| **Deprecated** \| **Index** \| **Help** | |  |
| **PREV**   **NEXT** | **FRAMES**    **NO FRAMES**     **All Classes** |  |


---
